# Diosgenin from *Dioscorea bulbifera*: Novel Hit for Treatment of Type II Diabetes Mellitus with Inhibitory Activity against α-Amylase and α-Glucosidase

**DOI:** 10.1371/journal.pone.0106039

**Published:** 2014-09-12

**Authors:** Sougata Ghosh, Piyush More, Abhishek Derle, Ajay B. Patil, Pramod Markad, Adersh Asok, Navanath Kumbhar, Mahemud L. Shaikh, Boppana Ramanamurthy, Vaishali S. Shinde, Dilip D. Dhavale, Balu A. Chopade

**Affiliations:** 1 Institute of Bioinformatics and Biotechnology, University of Pune, Pune, India; 2 Garware Research Centre, Department of Chemistry, University of Pune, Pune, India; 3 Centre for Research in Nanotechnology and Science, Indian Institute of Technology Bombay, Powai, Mumbai, India; 4 National Centre for Cell Science, University of Pune Campus, Ganeshkhind, Pune, India; 5 Department of Microbiology, University of Pune, Pune, India; University of Michigan Medical School, United States of America

## Abstract

Diabetes mellitus is a multifactorial metabolic disease characterized by post-prandial hyperglycemia (PPHG). α-amylase and α-glucosidase inhibitors aim to explore novel therapeutic agents. Herein we report the promises of *Dioscorea bulbifera* and its bioactive principle, diosgenin as novel α-amylase and α-glucosidase inhibitor. Among petroleum ether, ethyl acetate, methanol and 70% ethanol (v/v) extracts of bulbs of *D. bulbifera*, ethyl acetate extract showed highest inhibition upto 72.06 ± 0.51% and 82.64 ± 2.32% against α-amylase and α-glucosidase respectively. GC-TOF-MS analysis of ethyl acetate extract indicated presence of high diosgenin content. Diosgenin was isolated and identified by FTIR, ^1^H NMR and ^13^C NMR and confirmed by HPLC which showed an α-amylase and α-glucosidase inhibition upto 70.94 ± 1.24% and 81.71 ± 3.39%, respectively. Kinetic studies confirmed the uncompetitive mode of binding of diosgenin to α-amylase indicated by lowering of both Km and Vm. Interaction studies revealed the quenching of intrinsic fluorescence of α-amylase in presence of diosgenin. Similarly, circular dichroism spectrometry showed diminished negative humped peaks at 208 nm and 222 nm. Molecular docking indicated hydrogen bonding between carboxyl group of Asp300, while hydrophobic interactions between Tyr62, Trp58, Trp59, Val163, His305 and Gln63 residues of α-amylase. Diosgenin interacted with two catalytic residues (Asp352 and Glu411) from α-glucosidase. This is the first report of its kind that provides an intense scientific rationale for use of diosgenin as novel drug candidate for type II diabetes mellitus.

## Introduction

Diabetes mellitus is considered to be a severe complex multifactorial metabolic disorder characterized by hyperglycemia and abnormal carbohydrate metabolisms. It is associated with several complications like hyperlipidemia, retinopathy and cardiovascular diseases which are major causes of morbidity and death [1]–[3]. WHO has projected that by 2030 diabetes would become 7^th^ leading cause of death globally [4]. It is expected that diabetic population throughout the world would become 418 million by 2025 which will further increase till 552 million by 2030 [5], [6]. Presently, treatment of diabetes primarily involves reduction in hyperglycaemia by various groups of drugs like biguanides, thiazolidinediones, sulphonylureas, meglitinides and α-glucosidase inhibitors in addition to insulin [7]–[10]. However, due to unwanted severe side effects which are a major limitation, there is an increased demand for new antidiabetic agents [11]–[14]. Therefore, medicinal plants are thought to be a rich unexplored source of potent antidiabetic drugs [15]–[17]. However, lack of mechanism-based detailed *in-vitro* assays has posed a difficulty towards the scientific investigation of the same [18].

Traditional medicinal plants have served to be efficient antidiabetic agents for ages due to their rich diversity of phytochemicals. Thus, there lies a profound scope of discovery of new molecules with pharmacological significance towards management of type II diabetes mellitus (T2DM). Recently, we have demonstrated antidiabetic potential of *Dioscorea bulbifera* which is profusely used in Indian and Chinese system of traditional medicine owing to its anticancer, antioxidant, analgesic and anti-inflammatory properties [13], [19]. In our earlier reports, we have demonstrated that the excellent antioxidant property of the plant is attributed due to its unique phytochemistry [20]. Another strong evidence of the diversified uses of this plant system is its application in nanobiotechnology for synthesis of gold and silver nanoparticles of exotic size and shapes [21], [22]. Hereby *D. bulbifera* offers a great scope for discovery of molecules with pharmacological activity.

As a part of our growing interest for search of novel herbal antidiabetic agents, herein we have identified the active principle from *D. bulbifera* for pancreatic α -amylase inhibitory activity by bioactivity-guided fractionation. Hereby we report the isolation, structural elucidation, inhibitory activity and kinetics of the active component from *D. bulbifera* against pancreatic α-amylase and α-glucosidase. Using molecular docking studies with the aid of computational tool we have confirmed binding of active molecule to active sites of the enzymes.

## Materials and Methods

### Chemicals and Reagents

Petroleum ether, ethyl acetate, methanol and ethanol were procured from Qualigens, Mumbai, India. Dipotassium hydrogen phosphate (K_2_HPO_4_), potassium dihydrogen phosphate (KH_2_PO_4_), sodium potassium tartarate, sodium hydroxide (NaOH), porcine pancreatic *α*-amylase and sodium chloride (NaCl) was obtained from HiMedia Laboratories Mumbai, India. Acarbose was obtained from Bayer Pharmaceuticals Pvt. Ltd. (Mumbai, India). All the chemicals and reagents procured were of A.R. grade. Diosgenin, *α*-glucosidase, 4-nitrophenyl *α*-D-glucopyranoside and DNSA (dinitrosalicylic acid) were obtained from Sigma Aldrich, USA.

### Ethics Statement

Field sampling studies did not require specific permissions as all locations from where the plants were collected were not privately-owned or protected in any way and the field studies did not involve endangered or protected species. Entire procedure involving animals was carried out with guidelines of Institutional Animal Ethical Committee of National Centre for Cell Science, University of Pune Campus, Ganeshkhind, Pune-411007, India and all efforts were made to minimize suffering. The study was carried with prior approval (Project number EAF/2012/B-193) from Institute's Animal Ethics Committee (IAEC) of National Centre for Cell Science (NCCS).

### Plant material and preparation of extracts

*D. bulbifera* bulbs were collected from natural geographical landscapes of Western Ghats of Maharashtra, India, which were identified and authenticated by botanist from National Research Institute of Basic Ayurvedic Sciences, Central Council for Research in Ayurveda and Siddha, Department of Ayush, Ministry of Health and Family Welfare, Government of India, New Delhi, Nehru Garden, Kothrud, Pune, India assigning voucher specimen number 860. Extracts were prepared as per the process reported earlier [20]. In short, bulbs were washed, cut into pieces and shade dried followed by reduction to powder in an electric blender. 100 g of fine powder was cold extracted with 70% (v/v) ethanol in distilled water which was sequentially extracted with petroleum ether, ethyl acetate and methanol. Hydroalcoholic extract was subjected to lyophilization while petroleum ether, ethyl acetate and methanol extracts were evaporated to dryness under reduced pressure at 40 °C in rotary evaporator and were stored at 4°C in air-tight containers. Extracts were further reconstituted in DMSO (20%, v/v) to get a final concentration of 1 mg/mL which was used in all biochemical assays. Acarbose (1 mg/mL) was used as a reference standard in all the experiments.

### Isolation and characterization

In order to estimate the major compound and isolate the active principle, the extract showing maximum activity was initially subjected to GC-TOF-MS analysis as per our earlier report [20]. Approximately 1.5 g of crude extract showing maximum activity was fractionated on silica gel (60–120 mesh size) by column chromatography (4 cm × 20 cm) using a successive stepwise gradient of toluene: ethyl acetate (100∶0, 80∶20, 70∶30, 60∶40, 0∶100) as per the protocols reported for isolation of major components [23]. Each fraction was concentrated under reduced pressure at 40 °C. The bioactive fraction was loaded on a TLC plate (10 × 10 cm, Merck-60 F254, 0.25 mm thick) and developed using 30% ethyl acetate in toluene as mobile phase visualized by anisaldehyde sulphuric acid reagent followed by heating at 110 °C for 5 mins. The fractions showing similar patterns in high performance thin layer chromatography (HPTLC) were pooled together followed by careful monitoring of biological activity. FTIR was recorded on Shimazdu FTIR spectrometer. NMR spectra have been recorded with Varian 300 MHz spectrometer [24]–[26]. Pure bioactive sample was analyzed and compared with standard diosgenin by using Agilent Infinity series HPLC with eclipse C18 column (4.6 × 100 mm and 3.5 µm particle size). For this reverse phase chromatographic separation at isocratic mode with the mixture of acetonitrile: water (90∶10 v/v) was employed with a flow rate of 1 mL/min at 30°C. Changes in absorbance were measured at 214 nm using UV-Vis detector. This optimized HPLC method was scaled up on preparative HPLC: Shimdzu LC-8A preparative liquid chromatography with column phenomenex Luna 15u C18 (250 × 30 mm with 15micron particle size. Preparative HPLC purification afforded 60% yield. Purified bioactive compound isolated from preparative HPLC was then compared with the standard diosgenin sample by aforementioned HPTLC.

### Porcine pancreatic amylase inhibition assay

Chromogenic 3,5-dinitrosalicylic acid (DNSA) assay was employed to assess the α-amylase activity as reported earlier [27]. Isolated compound D (100 µg/mL) was incubated with 50 µg ml^−1^ of porcine pancreatic α-amylase at 37°C for 10 minutes [28]. One percent starch was used as substrate. α-amylase without D was used as control. Reducing sugar was estimated using DNSA assay at A 540 nm and the inhibitory activity was calculated by using the formula:
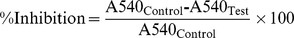



The mode of inhibition of PPA by D was determined by using Michaelis–Menten and Lineweaver–Burk equations [29]. Starch (1–5 mg ml^−1^) was incubated with D and PPA for 10 min and the residual enzyme activity determined by DNSA.

### Interaction studies by fluorescence and circular dichroism (CD) spectrometry

Fluorescence measurements of porcine pancreatic α - amylase were acquired using HORIBA JobinYvonFluorolog 3 model at 37 °C with a 1.0 cm path length within quartz cuvette in 0.02 M sodium phosphate buffer, pH 6.9 (containing 6 mM NaCl). Both excitation and emission slits were set at 3.0 nm. The samples were excited at 270 nm, and the emission spectra were recorded from 280 to 450 nm [30]. Concentration of D and enzyme used were same as above. CD spectra were recorded at 37 °C on a Jasco–J–815 spectropolarimeter at a scan speed of 40 nm/min with a response time of 1 s and a slit width of 1 nm. Quartz cell of 2 mm path length was used for the measurements in 190–300 nm range. All the measurements were made at a fixed enzyme vs D concentration mentioned above in phosphate buffered saline. Each spectrum reported is an average of three successive scans.

### Crude murine pancreatic amylase inhibition assay

10-week-old Swiss male mice weighing 20 gm were starved for 12 h. Pancreas was excised and homogenized with 10 mM ice cold phosphate buffer containing 6 mM NaCl (1∶10 dilution; w/v) supplemented with appropriate amount of protease inhibitors. Tissue homogenates were subjected to centrifugation for 10 min at 10,000 r.p.m. and the supernatant was considered as a source of enzyme that was diluted so as to get an absorbance of 0.4 at 280 nm [13]. Enzyme inhibition assay was carried out as described above. Percentage inhibition of the samples against pancreatic α-amylase was calculated.

### α-glucosidase inhibitory assays

Glucosidase inhibition assay of samples were carried out as per Ghosh *et al.*, 2012 [13]. 100 µL of α-glucosidase (0.1 unit/ml) was mixed with 200 µL of D (100µg/mL) and incubated for 1 hour at 37°C. Initiation of enzyme action was carried out by addition of of 10 mM *p*-nitrophenyl-α-D-glucopyranoside in 100 mM phosphate buffer of pH 6.8 and stopped by adding 2 mL of 0.1 M Na_2_CO_3_ after an incubation of 10 minutes at 37°C. α-glucosidase activity was determined by measuring absorbance of the *p*-nitrophenol released from *p*NPG at 420 nm using 96-well plate reader (SpectraMax M5, Molecular Devices Corporation, Sunnyvale, CA). One unit of glucosidase activity is defined as the amount of enzyme that hydrolyzed 1 µM of *p*-nitrophenyl pyranoside per minute under assay condition.
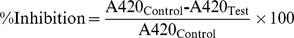



### Crude murine intestinal α-glucosidase inhibition assay

Intestinal extract of Swiss mice was prepared by the above mentioned process which was used as source of α-glucosidase. Inhibitory activity against the crude murine intestinal α-glucosidase was checked using *p*-nitrophenyl-α-D-glucopyranoside as substrate as per the above protocol.

### Computational methodology for molecular docking parameters

The crystal structure of receptor molecules (porcine pancreatic α-amylase, PPA and yeast glucosidase) were analyzed and downloaded from Protein databank (http://www.rcsb.org). The receptor molecules with high resolution were selected for molecular docking against diosgenin. Molecular docking studies were performed using AutodockVina 1.1.2 [31]. From the receptor molecules, non-polar hydrogen atoms were removed and Kollman united atom charges and polar hydrogen atoms were added. The obtained receptor structures were minimized for 5000 steps of steepest descent method using SpdbViewer to remove internal stain [32]. Minimized structures were finally subjected for docking studies. Molecular structure of ligand (diosgenin) was retrieved from Pubchem database (NCBI), and geometrically optimized by semi-empirical RM1 method using Spartan Pro 6.1.0 software [33], [34]. The Gasteiger charges and hydrogen atoms were added to the ligand molecule using AutdockVina. AutoGrid was used for calculating the grid maps and centered on the ligand binding site of PPA and yeast glucosidase, in such a way that it would totally cover the ligand molecule. The grid size was set to 25 A° × 25 A° × 25 with a grid spacing 0.375 A°. Docking results were visualized and analyzed using Ligpot and Pymol open source softwares [35].

## Results

### Isolation and identification of inhibitor

**Figure 1:**
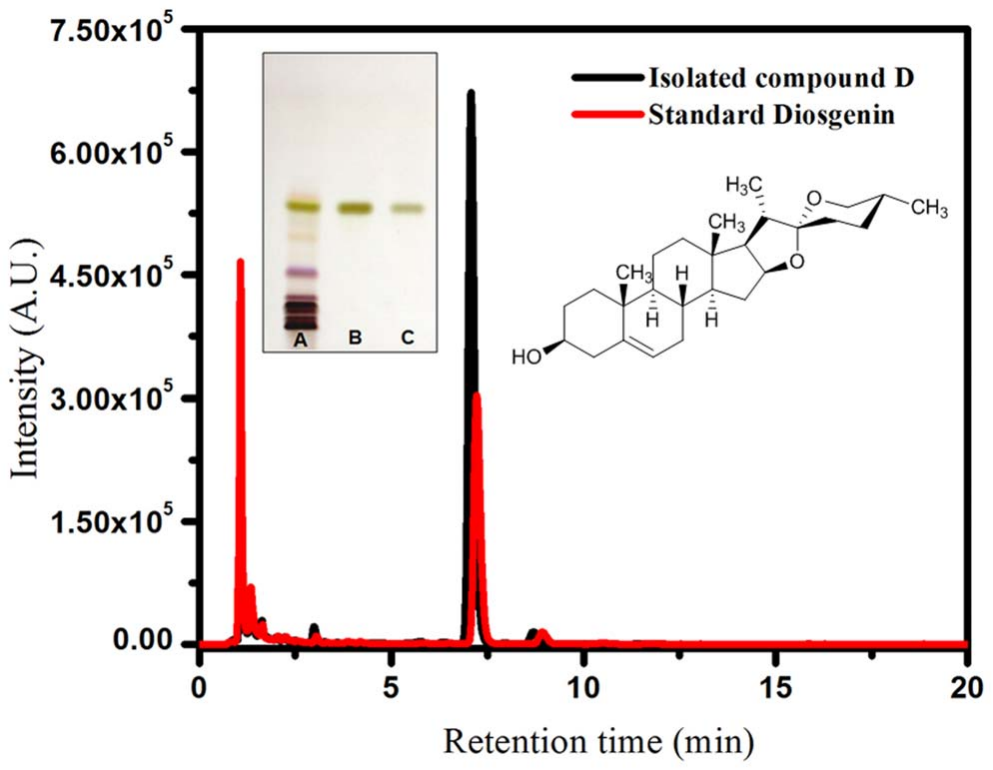
Characterization of purified compound D. HPLC profiles of standard diosgenin and isolated compound D exhibiting α-amylase and α-glucosidase inhibition. Inset left panel: HPTLC analysis of A) crude ethyl acetate extract of *D. bulbifera* bulb; B) isolated compound D and C) standard diosgenin. Inset right panel: Stereochemical structure of compound D as elucidated by NMR analysis identified as diosgenin.

Isolation of the bioactive compound was achieved by bioassay guided fractionation showing better inhibitory activity. Among 70% (v/v) ethanol extract (5.56 ± 0.51%, w/w), which was further fractionated sequentially yielding petroleum ether extract (0.65 ± 0.03%, w/w), ethyl acetate extract (0.78 ± 0.21%, w/w) and methanol extract (2.58 ± 0.22%, w/w), the results revealed that ethyl acetate extract of *D. bulbifera* bulbs showed potent inhibitory activity. GC-TOF-MS indicated the major phytoconstituent present in the ethyl acetate extract was diosgenin (Figures S1 and S2). Hereby, by column chromatography the bioactive fraction was eluted and confirmed by its prominent band in HPTLC with a *R_f_* value 0.42 (30% ethyl acetate in toluene) indicating its single compound purity. Further FTIR, ^1^H and ^13^C NMR based on the assignments of the chemical shifts (ppm) identified and confirmed the bioactive compound to be diosgenin (Figure S3, S4 and S5). HPLC analysis showed that the retention time for both isolated compound D and the standard diosgenin was 7.15 min. As shown in Figure 1, HPTLC of the HPLC purified compound D confirmed the single compound purity at *R_f_* value 0.42 similar to standard diosgenin, as compared to that crude starting material.

### Porcine pancreatic amylase inhibition

**Figure 2:**
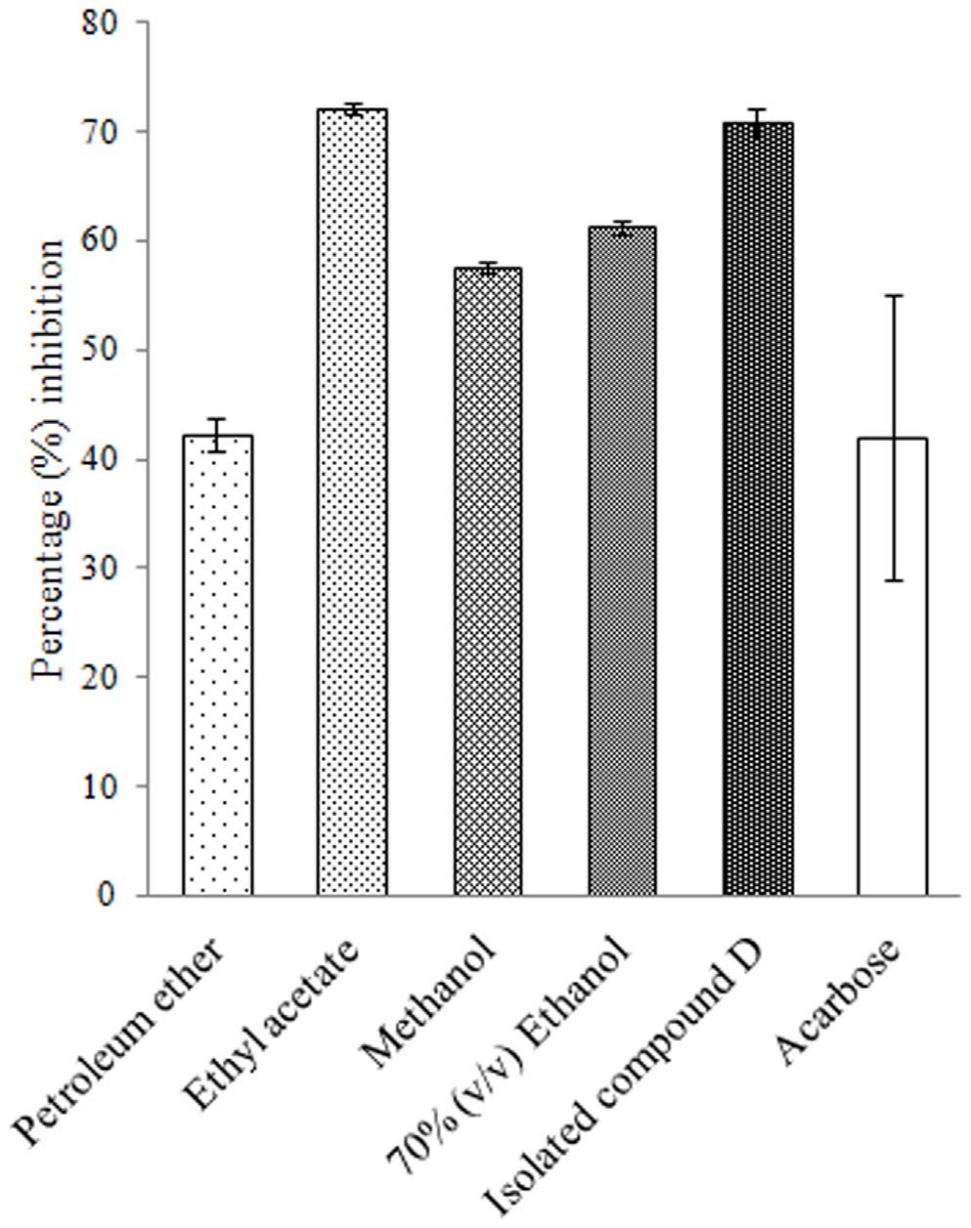
Percent *α*-amylase inhibition by plant extracts and isolated compound D. Acarbose is taken as standard inhibitor. The data is indicated as the mean SEM; [*n*  =  3].

**Figure 3:**
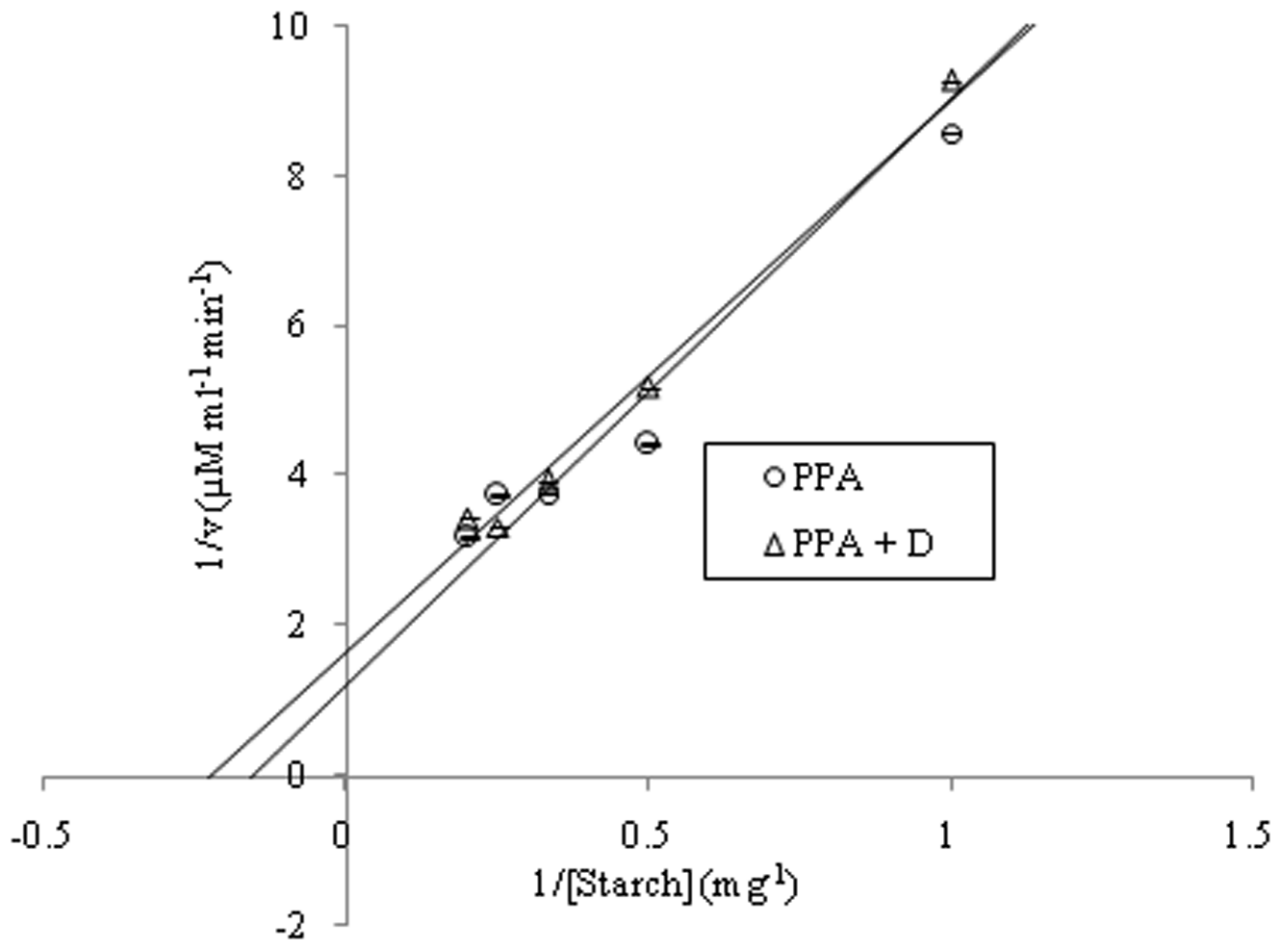
Kinetic analysis of porcine pancreatic α-amylase inhibition by isolated compound D by Lineweaver–Burk plot with starch as substrate.

Among all the extracts of *D. bulbifera* the ethyl acetate extract was found to show maximum inhibition upto 72.06 ± 0.51% followed by 70% ethanolic (v/v) extract which exhibited 61.26 ± 0.66% inhibition against porcine pancreatic α-amylase (Figure 2). However methanolic extract showed a lower inhibition of 57.47 ± 0.49% while petroleum ether extract showed an inhibition (42.24 ± 1.54%) comparable to acarbose. Compound D showed a superior inhibition of 70.94 ± 1.24% equivalent to ethyl acetate extract. Kinetic analysis revealed that the Km and Vm for the control uninhibited enzyme was 6.57 and 0.8 respectively (Figure 3). Both Km and Vm decreased to 4.47 and 0.6 respectively in presence of D indicating an uncompetitive mode of inhibition.

### Crude murine pancreatic amylase inhibition

**Figure 4:**
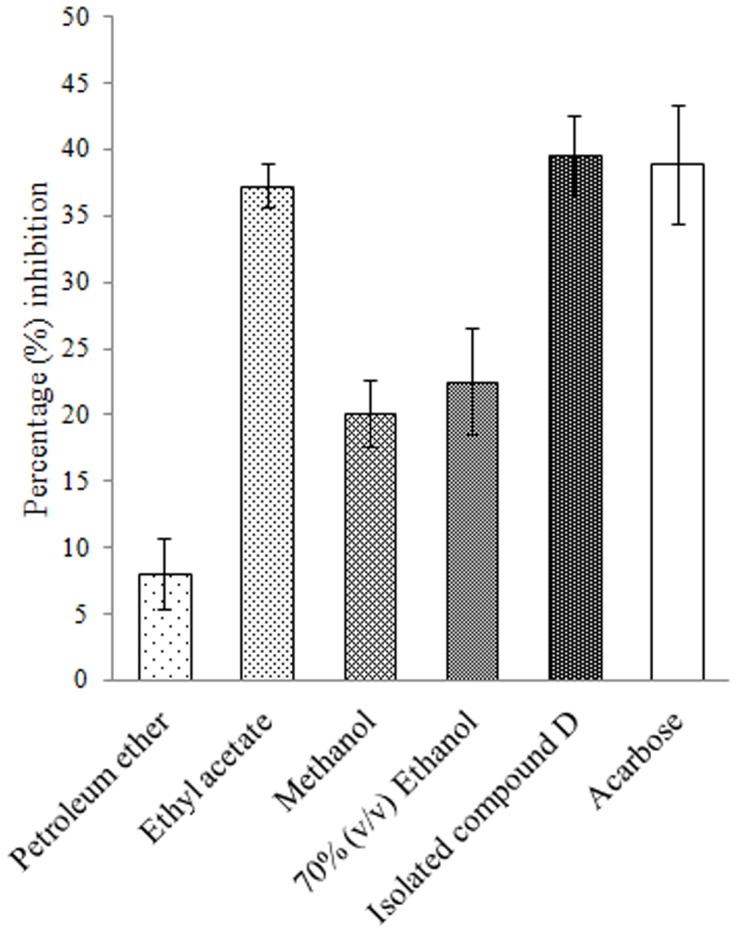
Percent crude murine pancreatic amylase inhibition by plant extracts and isolated compound D. Acarbose is taken as standard inhibitor. The data is indicated as the mean SEM; [*n*  =  3].

Ethyl acetate extract of *D. bulbifera* tuber showed the highest inhibition of 37.28 ± 1.6% while petroleum ether extract showed lowest upto 8.13 ± 2.67% (Figure 4). On the other hand 70% ethanol (v/v) extract (22.56 ± 4.03%) was found to exhibit superior activity as compared to methanolic extract (20.16 ± 2.54%). However, acarbose was found to show a higher percentage of inhibition upto 38.84 ± 4.45% while compound D showed an inhibition of 39.56 ± 3.02%.

### Fluorescence spectrometry and circular dichroism (CD) spectrometry

**Figure 5:**
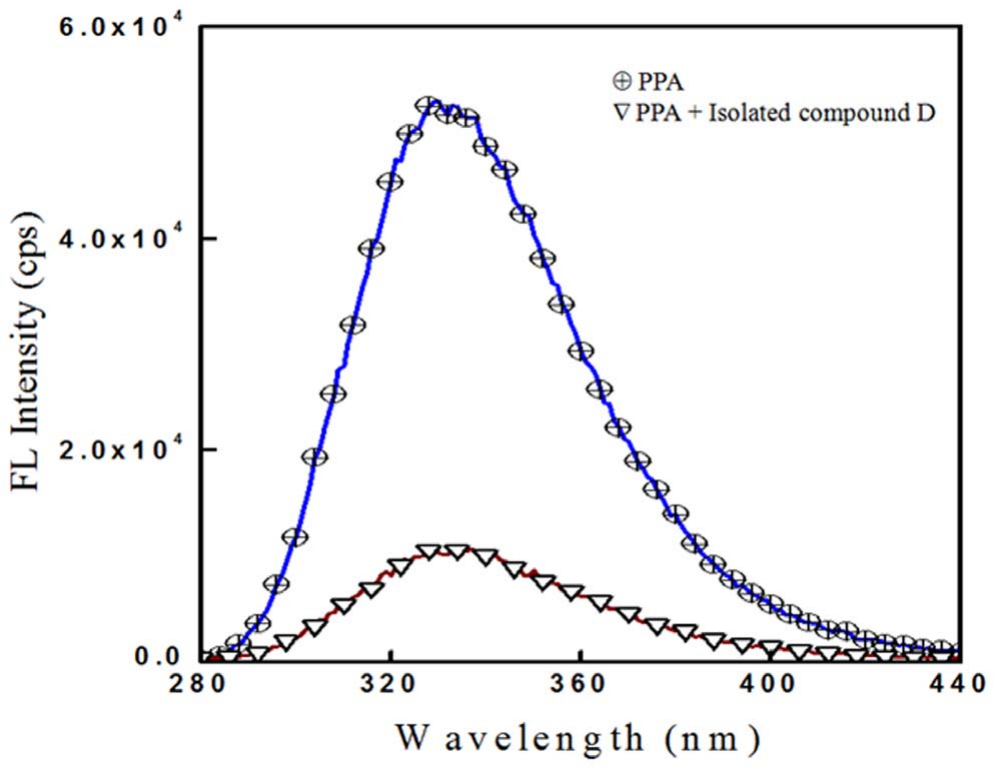
Quenching of intrinsic fluorescence of porcine pancreatic α-amylase bound to isolated compound D.

Interaction of D with α-amylase was confirmed by from the quenching of intrinsic fluorescence (Figure 5). Decay in fluorescence intensity of α-amylase in the presence of D was observed which could be attributed to dynamic interaction of D with the active site of α-amylase which may be the underlying principle of enzyme inhibition mechanism exhibited by D.

**Figure 6:**
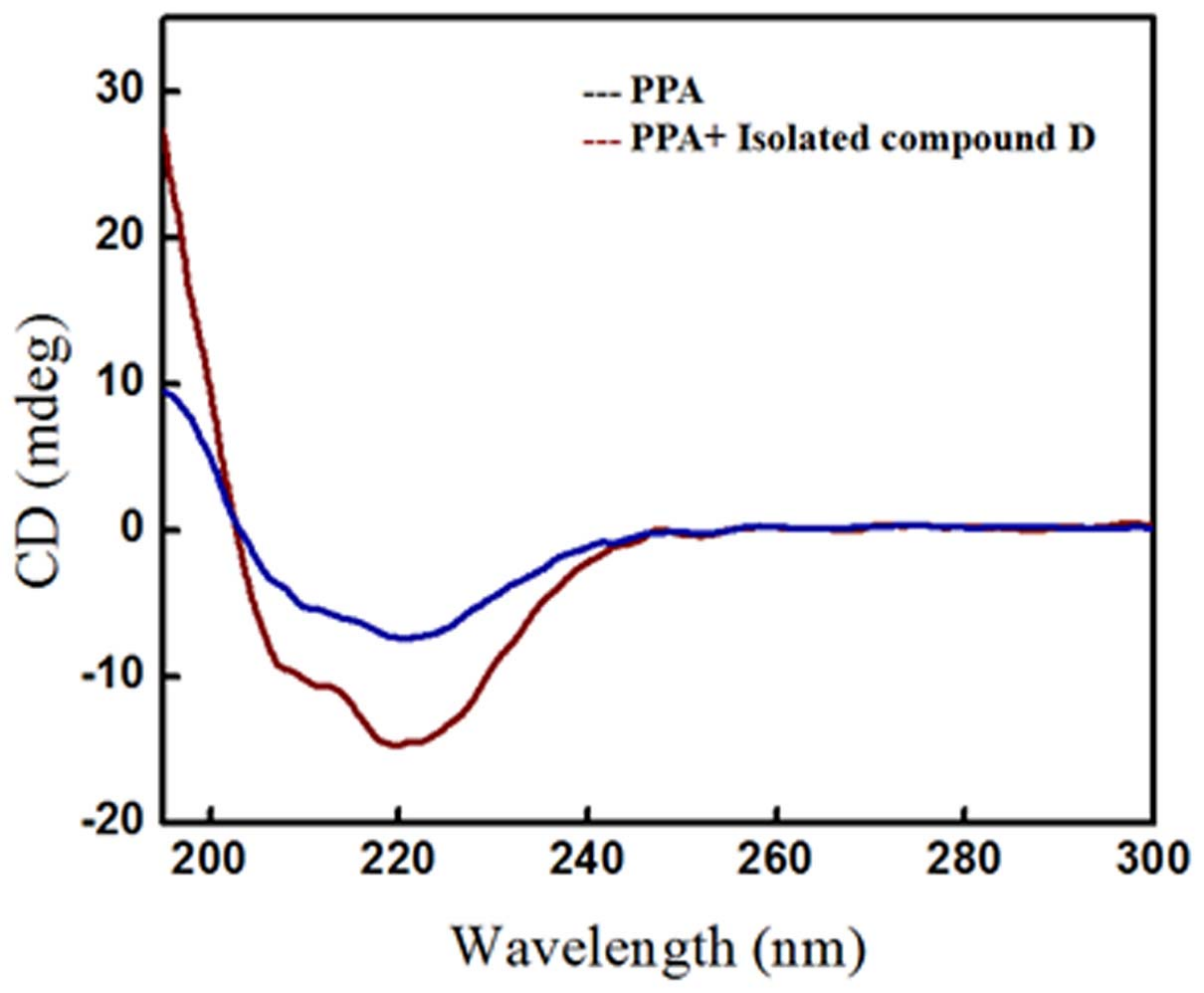
CD spectra of porcine pancreatic α-amylase bound with isolated compound D.

Circular dichroism (CD) studies indicated the changes in secondary structure of α-amylase confirming the interaction of D. A diminish in negative humped peaks at 208 nm and 222 nm in presence of D was observed when compared with the control enzyme (Figure 6).This clearly indicated a probable interaction of D with the active site of enzyme, resulting in conformational change in secondary structure of the enzyme. This observation along with our enzyme inhibition assay studies suggests a possible interaction of D with the active site of α-amylase.

### α-glucosidase inhibition

**Figure 7:**
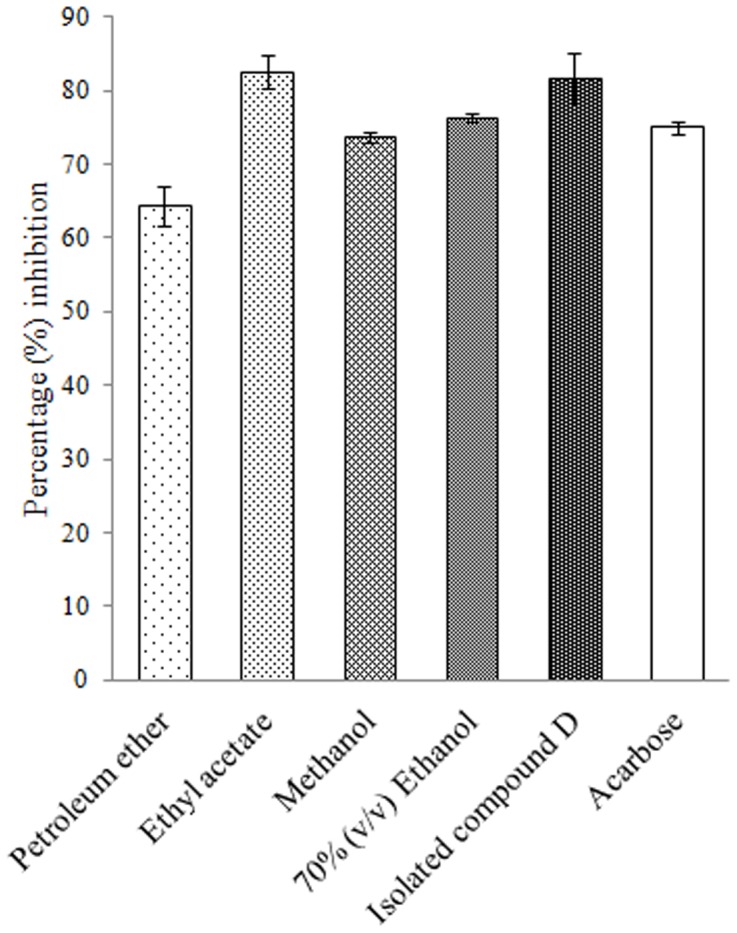
Percent α-glucosidase inhibition by plant extracts and isolated compound D. Acarbose is taken as standard inhibitor. The data is indicated as the mean SEM; [*n*  =  3].

Among all the extracts of *D. bulbifera* ethyl acetate extract (82.64 ± 2.32%) showed the most superior inhibitory activity against α-glucosidase enzyme followed by 70% ethanol (v/v) extract showing 76.25 ± 0.6% and methanol extract with 73.69 ± 0.8%. Petroleum ether extract showed lowest activity of 64.39 ± 2.66% (Figure 7). However, compound D showed a high activity of 81.71 ± 3.39% which was significant as compare to even acarbose.

### Crude murine intestinal glucosidase inhibition

**Figure 8:**
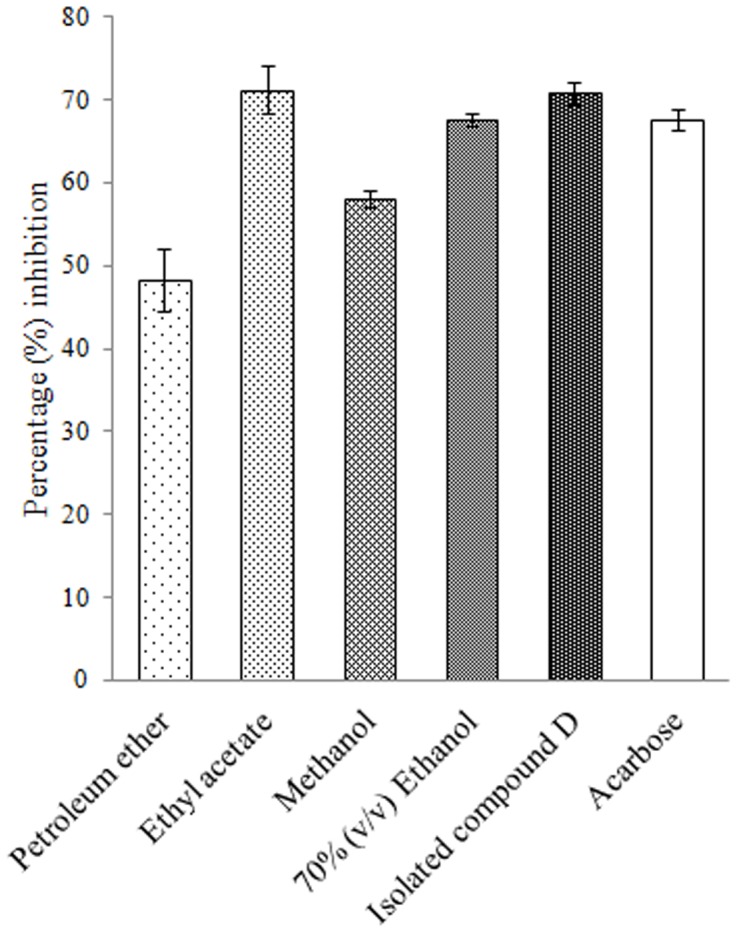
Percent crude murine intestinal glucosidase inhibition by plant extracts and isolated compound D. Acarbose is taken as standard inhibitor. The data is indicated as the mean SEM; [*n*  =  3].

Inhibitions of crude murine intestinal glucosidase were well in agreement with that of the pure α-glucosidase. In this study as well ethyl acetate extract of *D. bulbifera* tuber was found to exhibit most superior activity equivalent to 71.15 ± 2.9% followed by 70% ethanol (v/v) extract with 67.61 ± 0.77% (Figure 8). Petroleum ether and methanol extracts showed an inhibition of 48.24 ± 3.75% and 58.09 ± 0.92%, respectively. Compound D was found to show a superior activity of 70.78 ± 1.32% which was even higher than acarbose (67.61 ± 1.31%).

### Computational docking study for inhibition of porcine pancreatic α-amylase by diosgenin

**Figure 9:**
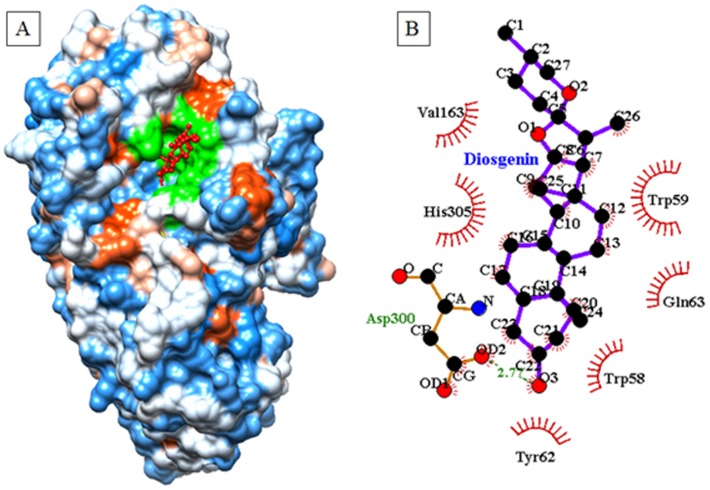
Binding of diosgenin to α-amylase active pocket. (a) Depicts docked conformation of diosgenin with porcine pancreatic α-amylase (1OSE.pdb), (b) hydrogen bonding and hydrophobic interactions from α-amylase and diosgenin inhibitor complex.

Eight docking runs were performed using the Lamarckian Genetic Algorithm. The generated docked conformations of receptor ligand complexes were analyzed for binding affinity. Structural alignments of receptor-ligand complexes with other available crystal structure were performed using Pymol software. Hydrogen bonding and hydrophobic interactions from α-amylase and yeast alpha glucosidase with diosgenin were visualized and analyzed by Ligplot **1.0**. Figure 9 depicted molecular docking results of α-amylase with diosgenin. The α-amylase-diosgenin inhibitor complex (Figure 9a) showed lowest energy (−7.4 kcal/mol) with highest binding affinity as compared with other generated α-amylase-diosgenin complexes. Selected inhibitor complex was analyzed for geometrical parameter such as hydrogen bonding, hydrophobic interactions and catalytic residues. The salient feature of α-amylase is presence of three highly conserved and catalytic residues such as Asp197, Glu233 and Asp300 in the active site pocket. Out of these, Asp300 is one of the catalytic residue which was observed to interact with diosgenin molecule in molecular docking study (Figure 9b). The carboxyl group of Asp300 is involved in strong hydrogen bonding interaction with oxygen O3 of diosgenin having bond distance 2.77A°. Besides this, some hydrophobic interactions were also observed between Tyr62, Trp58, Trp59, Val163, His305 and Gln63 residues with diosgenin (Figure 9b). All these interactions (Figure 9b) are important for positioning of diosgenin in catalytic pocket in order to inhibit the activity of α-amylase. Molecular docking results showed close resemblance with experimental data.

### Computational docking study for inhibition of α- glucosidase with diosgenin

**Figure 10:**
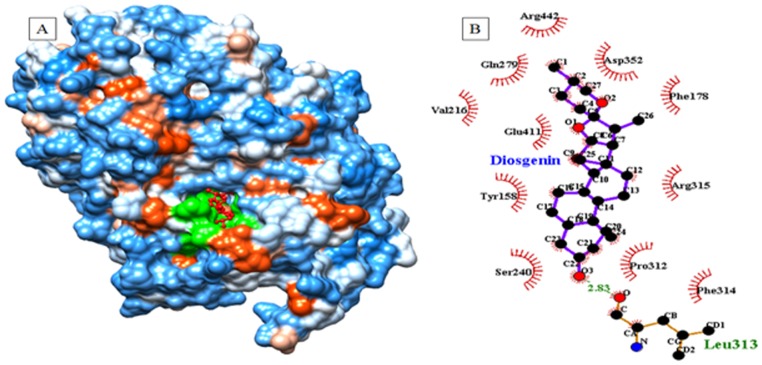
Binding of diosgenin to α-glucosidase active pocket. (a) Depicts docked conformation of diosgenin with yeast alpha glucosidase (3AXI.pdb), (b) hydrogen bonding and hydrophobic interactions from alpha glucosidase-diosgenin inhibitor complex.

Similarly, we have determined inhibition mechanism for yeast α-glucosidase by diosgenin using molecular docking study. Above discussed computational procedure was adopted for selection of stable receptor-ligand inhibitor complex within generated docking conformations. Analyses were also made for geometrical parameters such as hydrogen bonding, hydrophobic interactions and interactive catalytic residues from α-glucosidase-diosgenin inhibitor complex. Figure 10a showed lower energy (−9.7 kcal/mol) α-glucosidase-diosgenin complex with highest binding affinity due to presence of hydrogen bonding and hydrophobic interactions. Three highly conserved and catalytic residues such as Asp197, Glu233 and Asp300 from glucosidase family were reported using crystallographic studies. In accordance with crystal structure data, interactions of two catalytic residues Asp352 and Glu411 with diosgenin was observed in docking studies (Figure 10b). These residues were preferably involved in positioning of diosgenin molecule within the active sites pocket of α-glucosidase (Figure 10a). Other residues such as Ser240, Tyr158, Val216, Gln279, Arg442, Phe178, Arg315, Pro312 and Phe314 have also been involved in hydrophobic interactions with diosgenin (Figure 10b). Along with catalytic residues, Leu313 is also involved in strong hydrogen bonding interaction (2.83A°) with oxygen (O3) of diosgenin. This interaction may provide further stability to α-glucosidase-diosgenin complex (Figure 10b). All of these interacting residues help diosgenin to fit well in the active site pocket of α-glucosidase. Thus, hydrogen bonding and hydrophobic interactions from α-glucosidase might be involved in hydrolysis of substrate molecules. The molecular docking results of α-glucosidase are in close agreement with experimental data, in respect to inhibition activity by diosgenin.

## Discussion

One of the most powerful strategies for controlling post-prandial hyperglycaemia in case of diabetes mellitus is inhibition of the starch hydrolyzing enzymes. Due to the notable side effects of the drugs researches have been now focused on many herbal extracts which were mentioned and used in traditional medicine. However, such medicinal plants failed to gain much importance due to lack of scientific evidence and poorly understood phytochemistry and their mechanism of action. The spectacular success of our recent report on antidiabetic effect of crude extracts of *D. bulbifera* inspired us to decipher the mechanism of action of the principle bioactive component in the extract [13]. A recent preliminary report showed extracts of *D. bulbifera* to exhibit antihyperglycemic activity in streptozotocin treated Wistar rats. [36].

In this study we found that extracts of *D. bulbifera* could significantly inhibit α-amylase and α-glucosidase which provides a detailed mechanism underlying glucose lowering effect of the plant extracts. Bioactive fraction of ethyl acetate extract was rich in diosgenin content which we have previously reported as 94.05%. [20]. The active principle was identified to be diosgenin which is considered as a major phytoconstituent of *D. bulbifera* [37]–[39]. Diosgenin is reported to have immense therapeutic potential to cure diseases like dyslipidemia/obesity, inflammation, liver function and cancer [40]–[46]. Our study has added up one more attribute to the intense pharmacological significance of diosgenin towards the management of T2DM. Diosgenin exhibited a significant level of α-amylase inhibitory effect which supports its role on reduction of high blood glucose levels. It was reported that diosgenin exhibited a significant glucose lowering effect after supplementation in Wistar rats [47]. Similar *in-vivo* studies have also demonstrated that diosgenin from *D. esculenta* (lesser yam) control hyperglycemia in the type 1 diabetes model rats through an increase muscular GLUT4 translocation, as well as increased phosphorylation of Akt and PKC ζ/λ supporting the fact that diosgenin-induced dehydroepiandrosterone (DHEA) plays a key role in control of hyperglycemia by activating muscular GLUT4 signaling pathway [48]. Thus, our study provides a strong rationale to the antidiabetic property of diosgenin by enzyme inhibition which is well in agreement with the property of diosgenin for altering various intestinal enzymes in streptozotocin induced diabetic male Wistar rats [49]. Diosgenin showed an excellent inhibitory activity against α-amylse which was confirmed by the interaction studies. The active site of α-amylase contains tryptophan (Trp) residues in the β2– α2 loop of the catalytic (β/α)_8_ barrel which contributes to a strong intrinsic fluorescence [50]. Thus the interaction of diosgenin with α-amylase active site leading to quenching of intrinsic fluorescence, indicated its direct interaction with Trp residue [51]. Alternatively, circular dichroism (CD) studies revealed the nature of interaction of α-amylase with diosgenin. As a first step, CD spectroscopy provides the information of detailed secondary structure of enzyme [52]. Two peaks minima of α-amylase at 208 and 222 nm, are attributed to a high α-helical content of enzyme [53]. Any alteration in the conformational changes of α-amylase can be reflected in CD spectra, either as a blue shift or diminished minimum. A diminish in negative humped peaks at 208 nm and 222 nm in presence of diosgenin clearly indicated the interaction of diosgenin with of enzyme, resulting in conformational change in secondary structure of the enzyme.

Computational docking studies provided immensely important information towards understanding the mechanism behind active site binding interactions [25], [26]. The salient feature of α-amylase is presence of three highly conserved and catalytic residues such as Asp197, Glu233 and Asp300 in the active site pocket [54]. Similar types of hydrogen bonding and hydrophobic interactions have also been discussed in earlier crystal structures of α-amylase with different inhibitor molecules [55]–[57]. In accordance with fluorescence and CD studies the docking interaction showed involvement of tryptophan residue in catalytic pocket of α-amylase as reported in crystallography data [55]–[57]. Similarly, a high enzyme inhibition against yeast α-glucosidase was observed experimentally as compared with α-amylase. Docking results are in close agreement with reports with earlier crystal structure data of α-glucosidase from *Saccharomyces cerevisiae* and *Geobacillus sp.* [58], [59]. α-glucosidase-diosgenin inhibitor complex showed lowest energy (−9.7 kcal/mol) with highest binding affinity as compared to α-amylase-diosgenin inhibitor complex (−7.4 kcal/mol). Diosgenin interacts with two catalytic residues (Asp352 and Glu411) from α-glucosidase, which enable to produce lowest energy inhibitor complex while only one catalytic residue (Asp300) from α-amylase is involved in hydrogen bonding interaction with diosgenin. In addition to hydrogen bonding interactions, other hydrophobic interactions helped to produce highest binding affinity of diosgenin towards the α-glucosidase. Above discussed computational results are in close agreement with the mechanism of action proposed for amylase-acarbose and other inhibitor molecules [55]–[58], [60]. The predicted molecular docking complexes may help to understand the mechanism of α-amylase and α-glucosidase inhibition by diosgenin. Hence, computational study along with experimental validation may help to design a new drug candidate of natural origin against diabetes providing an intense rationale for application of diosgenin as a lead drug candidate for the treatment of T2DM.

## Conclusion

*D. bulbifera* has significant applications as traditional herbal medicine for treatment of diabetes indicating the scope to find a mechanism of the principle component and validation of its target of action. Hereby, for the first time we report bioassay guided isolation of the active principle which was identified to be diosgenin. Diosgenin exhibited potent inhibition against both porcine pancreatic α-amylase and α-glucosidase as well as against crude murine amylase and glucosidase. Mode of binding was established to be uncompetitive inhibition that was strongly supported by detailed interaction studies and advanced computational docking. Thus, herein we add one more attribute to the spectrum of pharmacological significance of diosgenin supporting its promises to be a lead candidate in managing T2DM.

## Supporting Information

Figure S1 **GC-TOF-MS chromatogram of ethyl acetate extract of** ***D. bulbifera*** **bulb.**
(DOC)

Figure S2 **Mass spectra of isolated compound D identified by GC-TOF-MS as diosgenin.**
(DOC)

Figure S3 **FTIR spectra of isolated compound D.**
(DOC)

Figure S4 **^1^H NMR (300MHz, CDCl_3_) of isolated compound D.**
(DOC)

Figure S5 **^13^C NMR (75MHz, CDCl_3_) of isolated compound D.**
(DOC)
