# Supplementary figures and images for: Diosgenin from Dioscorea bulbifera: Novel Hit for Treatment of Type II Diabetes Mellitus with Inhibitory Activity against α-Amylase and α-Glucosidase
Source: PLoS One. 2014 Sep 12;9(9):e106039. doi: 10.1371/journal.pone.0106039 (PMC4162539; doi:10.1371/journal.pone.0106039)

**
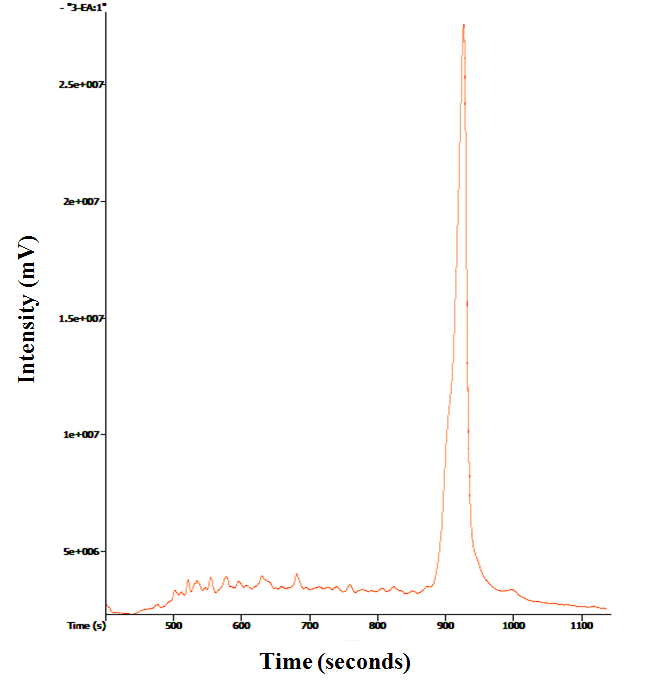
**

Figure S1. GC-TOF-MS chromatogram of ethyl acetate extract of *D. bulbifera* bulb.

Supplement: Figure S1 — GC-TOF-MS chromatogram of ethyl acetate extract of D. bulbifera bulb. (DOC) [file pone.0106039.s001.doc]

**
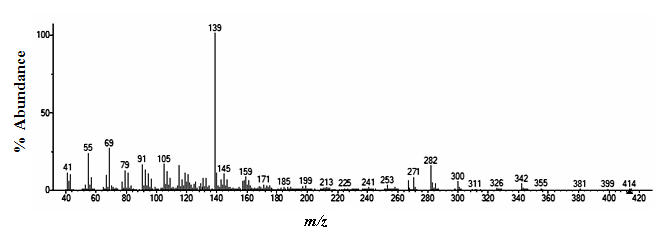
**

Figure S2. Mass spectra of isolated compound D identified by GC-TOF-MS as diosgenin.

Supplement: Figure S2 — Mass spectra of isolated compound D identified by GC-TOF-MS as diosgenin. (DOC) [file pone.0106039.s002.doc]

**
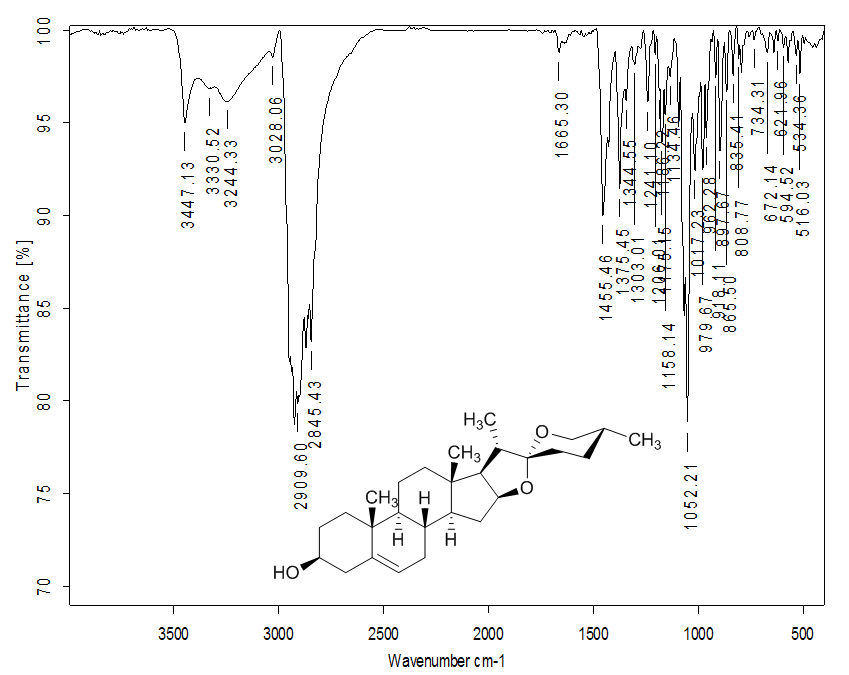
**

Figure S3. FTIR spectra of isolated compound D.

Supplement: Figure S3 — FTIR spectra of isolated compound D. (DOC) [file pone.0106039.s003.doc]

**
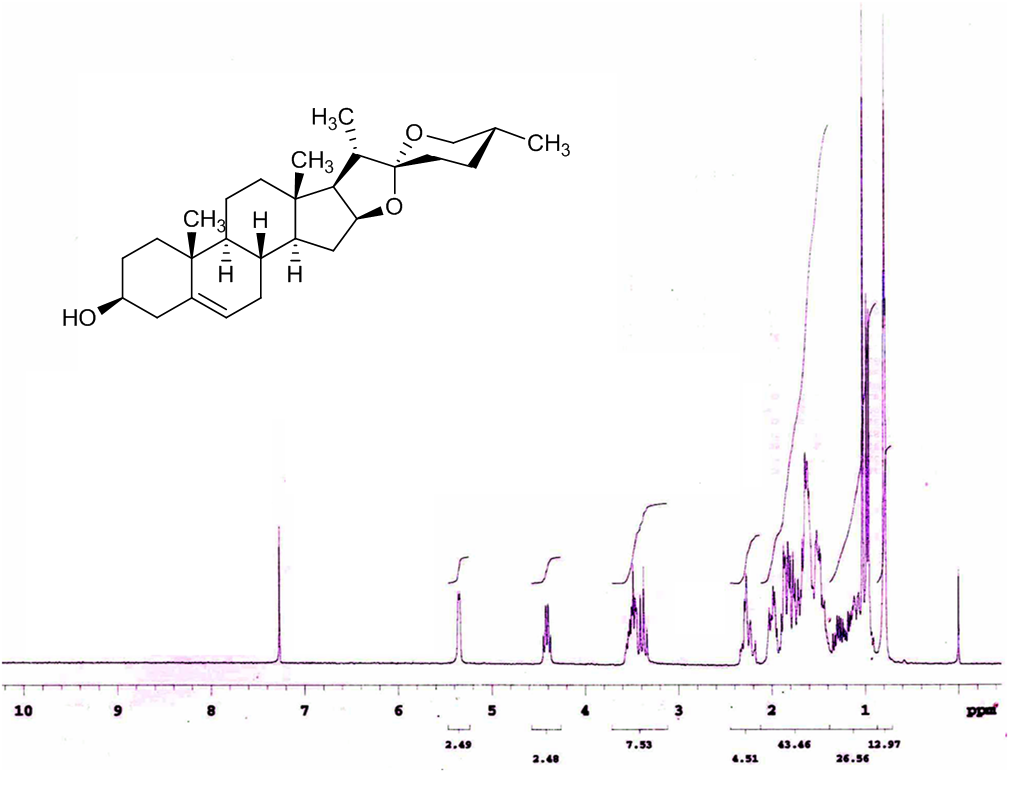
**

Figure S4. 1H NMR (300MHz, CDCl3) of isolated compound D.

Supplement: Figure S4 — 1H NMR (300MHz, CDCl3) of isolated compound D. (DOC) [file pone.0106039.s004.doc]

**
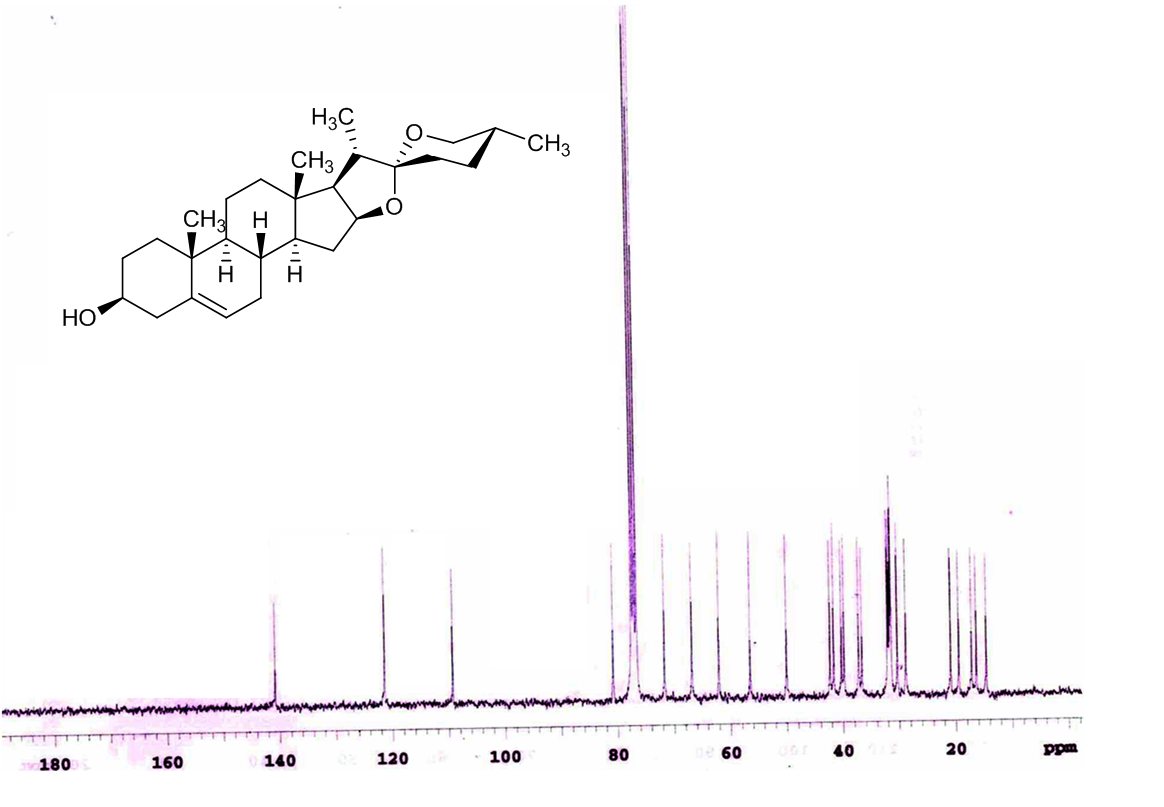
**

Figure S5. 13C NMR (75MHz, CDCl3) of isolated compound D.

Supplement: Figure S5 — 13C NMR (75MHz, CDCl3) of isolated compound D. (DOC) [file pone.0106039.s005.doc]
